# Evaluating the use of hair as a non-invasive indicator of trace mineral status in woodland caribou (*Rangifer tarandus caribou*)

**DOI:** 10.1371/journal.pone.0269441

**Published:** 2022-06-28

**Authors:** Naima Jutha, Claire Jardine, Helen Schwantje, Jesper Mosbacher, David Kinniburgh, Susan Kutz

**Affiliations:** 1 Department of Pathobiology–Ontario Veterinary College, University of Guelph, Guelph, Ontario, Canada; 2 Canadian Wildlife Health Cooperative—Ontario Veterinary College, University of Guelph, Guelph, Ontario, Canada; 3 Ministry of Forests, Lands, Natural Resource Operations, and Rural Development–Wildlife and Habitat Branch, Government of British Columbia, Nanaimo, British Columbia, Canada (Emeritus status); 4 Department of Ecosystem and Public Health–Faculty of Veterinary Medicine, University of Calgary, Calgary, Alberta, Canada; 5 Alberta Centre for Toxicology, University of Calgary, Calgary, Alberta, Canada; Universitat Autonoma de Barcelona, SPAIN

## Abstract

Trace mineral imbalances can have significant effects on animal health, reproductive success, and survival. Monitoring their status in wildlife populations is, therefore, important for management and conservation. Typically, livers and kidneys are sampled to measure mineral status, but biopsies and lethal-sampling are not always possible, particularly for Species at Risk. We aimed to: 1) determine baseline mineral levels in Northern Mountain caribou (*Rangifer tarandus caribou*; Gmelin, 1788) in northwestern British Columbia, Canada, and 2) determine if hair can be used as an effective indicator of caribou mineral status by evaluating associations between hair and organ mineral concentrations. Hair, liver, and kidney samples from adult male caribou (n_Hair_ = 31; n_Liver_, n_Kidney_ = 43) were collected by guide-outfitters in 2016–2018 hunting seasons. Trace minerals and heavy metals were quantified using inductively-coupled plasma mass spectrometry, and organ and hair concentrations of same individuals were compared. Some organ mineral concentrations differed from other caribou populations, though no clinical deficiency or toxicity symptoms were reported in our population. Significant correlations were found between liver and hair selenium (rho = 0.66, p<0.05), kidney and hair cobalt (rho = 0.51, p<0.05), and liver and hair molybdenum (rho = 0.37, p<0.10). These findings suggest that hair trace mineral assessment may be used as a non-invasive and easily-accessible way to monitor caribou selenium, cobalt, and molybdenum status, and may be a valuable tool to help assess overall caribou health.

## Introduction

As we continue to see nationwide declines in caribou populations, uncertainty surrounding the underlying mechanisms responsible remains a significant management challenge. Trace minerals, elements required in minute amounts for normal physiological function, are critical to many biochemical pathways and are essential for life in all species [1, 2]. Even subtle imbalances in trace minerals can have major downstream metabolic effects, with deficiencies and overloads resulting in impaired cell function or cellular damage [2]. In ruminants, deficiencies of copper or selenium can cause clinical syndromes such as abnormal hoof keratinization and degenerative nutritional myopathy (white muscle disease), respectively, as well as decreased reproductive success and increased susceptibility to bacterial infections [3–6]. Similar effects of mineral imbalances are assumed to occur in wild ungulates and, additionally, imbalances in trace minerals may contribute to population declines [1, 7, 8]. Constraints on dietary trace mineral access by caribou may even influence migration patterns, reproductive success, and calf survival [9]. Excessive amounts of some trace minerals, as well as heavy metals such as cadmium and lead, are toxic in domestic and wild ungulates, and can cause clinical disease, reduced immune function, and even impaired fecundity and population productivity [3, 5, 10]. It follows that the monitoring of trace mineral concentrations should be an important component of assessing individual and population health status trends in caribou and free-ranging wildlife in general.

Quantifying trace minerals levels in storage organs (liver and kidney) is currently considered best practice for understanding the current status in an individual. This approach in wildlife requires either invasive biopsies or lethal sampling, which becomes particularly difficult to justify when dealing with Species at Risk such as caribou. In live-captured animals, the standard method generally involves measurement of trace mineral concentrations in blood or serum, but correlations with liver and/or kidney values tend to be poor for most minerals [11, 12]. Trace mineral concentrations can also be quantified in hair, however, the relationships between levels in hair and those in livers and kidney are not well understood, nor have ‘normal’ levels been established, leading to ambiguity of how this metric should be interpreted [1, 13, 14]. Additionally, season, sex, age, and other confounding factors can influence the deposition of minerals into hair, and thus complicate analyses and interpretation [13]. That said, collection of hair is already incorporated into many wildlife health monitoring programs and it is used for various purposes, such as radioisotope analysis, DNA extraction, or hormone analysis [15–18]. Hair is relatively simple to collect, and it is easily stored at room temperature or frozen. Additionally, hair growth occurs in a defined time period after which it is separated from metabolic activity of the body [13], and so it provides a record of the physiological status of the animal at the time of hair growth.

In this study, we evaluated the relationships between key mineral concentrations in hair and those in liver and kidney tissue and, concurrently, determined the trace mineral and heavy metal status of Northern Mountain caribou (*Rangifer tarandus caribou* Gmelin, 1788) from northwestern British Columbia, Canada. Samples were collected through an outfitter-based wildlife health sampling program, so animals were consistently fall-hunted, adult, male caribou, with hair samples being taken near the end of the natural growth phase. As an ecotype designated *special concern* in the Species at Risk Act [19, 20], with little available data on health and population status [21], reliable, non-invasive methods of health and population monitoring are vital. In our study, the consistency in the demography of sampled animals, timing of sample collection, and access to paired hair and organ samples supported a critical evaluation of the use of hair trace minerals as indicators of individual and population health in caribou and free-ranging wildlife in general.

## Methods

### Study population and sample collection

**Fig 1:**
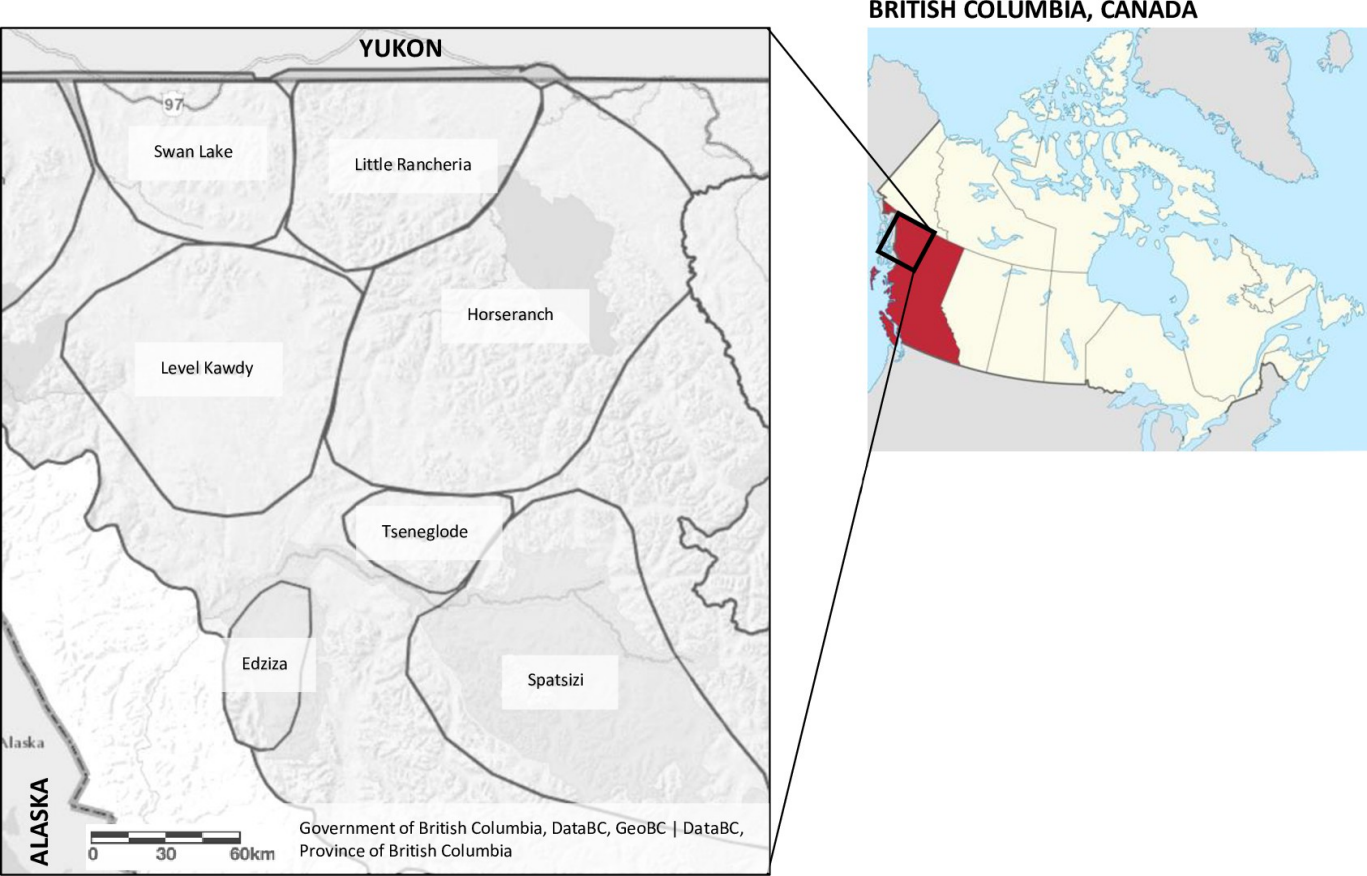
Study area: The Tahltan nation traditional territory with herd boundaries of 7 Northern Mountain woodland caribou herds included in this study.

We studied 7 herds of the Northern Mountain ecotype of woodland caribou (*R*. *t*. *caribou*) occurring in the traditional territory of the Tahltan Nation (northwestern British Columbia, Canada) (Fig 1). No animals were killed for the purposes of this study. Caribou were legally hunted in their natural habitat by authorized non-resident hunters during the fall hunting season (15 August– 15 October), accompanied by guide-outfitter members of the Tahltan Guide and Outfitters Association (TGOA), per regulations outlined in the B.C. Hunting and Trapping Synopsis under the Wildlife Act (Government of British Columbia). Samples were contributed by guide-outfitters participating in a harvest-based wildlife health sampling program initiated in 2016 and use for this study was approved per the BC Wildlife Permit MRSM 18–285261 (Government of British Columbia Ministry of Forests, Lands, Natural Resource Operations, and Rural Development (FLNRORD)) and the Animal Use Protocol AC-18-0093 (University of Calgary Animal Care Committee).

The study population included adult male Northern Mountain Caribou hunted by guided hunters in the fall of 2016, 2017, and 2018. Sixty-three sample kits were collected from animals hunted between 25^th^ August and 11^th^ October from 2016 to 2018. Participating guide-outfitters collected a standard set of samples and data from harvested animals [18]. This included hair from the dorsal shoulder region of the harvested animal, a 10 cm^2^ section of skin from the dorsal rump area, samples of liver tissue (~ 5 cm^3^ section), and whole left kidneys. In some cases, participants submitted partial left kidneys rather than the entire organ. Samples were stored in Whirl Pak™ sterile sample bags and frozen at -20°C until processing and analysis. Central incisors were submitted to Matson’s Laboratory, Manhattan, Montana for aging by cementum ring analysis [22].

### Sample analysis

#### Hair analysis

Hair from the shoulder was preferentially used. In cases where insufficient sample was collected, hair from rump skin sections of the same individual was collected in the lab by shaving as close to the skin surface as possible. Visible debris such as soil and vegetation were removed from hair samples using plastic forceps. Samples were then washed twice in 96% ethanol and ultrapure Type 1 reagent-grade water to remove further external contamination then placed in clean paper envelopes and oven-dried at 50°C for at least 24 hours. 30–50 mg of dried hair was weighed and added to 2 mL of 70% HNO3 in a plastic vial (TMF Vessel, 100mL; Milestone, Shelton, CT, USA). The vials were closed with air-tight caps and digested using a high-pressure microwave reactor (ETHOS EZ Microwave Digestion System; Milestone, Shelton, CT, USA). The temperature in the reactor was gradually increased from room temperature to 220°C over one hour, and then gradually cooled to room temperature over one hour. 2 mL of each digested sample was transferred to a falcon tube and diluted with ultrapure Type 1 water to a total volume of 4 mL and stored at 5°C until analysis. Each sample was further diluted with Type 1 water to a final dilution of 1:10 and hair mineral concentrations were determined using high-resolution inductively coupled plasma mass spectrometry (ICP-MS, 8800 Triple Quadrupole ICP-MS, Agilent) at the Alberta Center for Toxicology, University of Calgary. Instrument calibration verification for Quality Assurance (QA) were performed before, during, and after sample analyses using certified reference materials (Trace Elements in Natural Water (NIST1640a); Multi-Element Standard (SCP Science); and Environmental Calibration Standard (Agilent)). For each digestion (15 vials per run), 1 sample was a blank sample containing only acid to check for any contamination in laboratory procedure; 1 sample consisted of reference material for QA; and 13 vials contained samples. Of these samples, 1 sample was randomly selected to be run in duplicate for QA for each run. A maximum deviation limit of 20% between duplicates was set for the results in the run to be accepted, and all samples run in duplicate met these criteria when amount of mineral detected was greater than the method Limit of Quantitation (LOQ). For samples run in duplicate, the average of the two mineral concentration values was used for analysis. The LOQ (wet weight, digested sample) for Co, Pb, and Mo was 0.005 mg/L, for Mn and Se was 0.001 mg/L, for Cd, Cu, Zn was 0.005 mg/L, and for Fe was 0.05 mg/L. Mineral concentrations detected but falling below LOQ were included in the analysis. In cases where concentrations fell below detection limits, values of half the detection limit were assigned to assess correlations [23], and omitted for reporting baseline hair concentrations. Quality assurance was further confirmed in each batch, using certified reference materials (NIST2976 freeze-dried mussel tissue, National Institute of Standards and Technology; and NRC DORM-4 “Fish Protein Certified Reference Material for Trace Metals”, National Research Council Canada) as positive controls, and blank samples as negative controls. Blanks were negligible for all samples, and the laboratory positive controls were measured within acceptable ranges of certified reference values for all elements studied. Results are reported in mg/kg dry weight.

#### Kidney and liver tissue analysis

The outermost portions of liver samples and partial kidney samples were removed to minimize external trace mineral contamination from handling. Sterile stainless-steel scalpel blades were used, with efforts made to minimize instrument use where possible. Extra tissues and the renal capsule were removed from kidneys. When only partial kidney samples were submitted, those samples with unequal cortex:medulla ratios were discarded. Samples (minimum 5 g tissue, wet weight) were submitted to a commercial laboratory (ALS Environmental, Vancouver, BC). Metals analysis was conducted as described in by Horvath [24], where tissues were homogenized and then subsampled prior to being hot block digested with nitric and hydrochloric acids combined with hydrogen peroxide. Analysis for tissue concentrations of various trace minerals was done by collision cell—inductively coupled plasma—mass spectrometry (CC-ICP-MS), modified from the standard US-EPA Method 6020A [25]. Moisture content of tissues (% Moisture) was determined gravimetrically by drying each sample at 105°C for a minimum of 6 hours.

#### Samples were run in duplicate

A maximum deviation of 20% between duplicates was applied to qualify samples to be included in data analysis, and all samples met these criteria. Quality assurance was confirmed in each batch, using certified reference materials (NRC DORM-4 “Fish Protein Certified Reference Material for Trace Metals”, National Research Council Canada) and laboratory control samples as positive controls and method blank samples as negative controls. Blanks were negligible for all samples, and the concentrations measured were within acceptable ranges of certified reference values for all elements, meeting data quality objectives set by the commercial laboratory. In cases where concentrations fell below detection limits, values of half detection limit were assigned [23]. Results are reported in mg/kg dry weight (DW).

### Statistical analysis

Liver and kidney trace mineral concentrations were compared to those published for other Canadian caribou herds/ecotypes using t-tests with Bonferroni multiple comparisons corrections. Shapiro-Wilk tests and visualization of residual plots were used to assess normality and homoscedasticity in the dataset. Where necessary and possible, data were logarithmically transformed to meet normality assumptions. Spearman rank correlations between hair, liver, and kidney mineral concentrations were assessed (Tables 2 and 3). The Cook’s distance test was used to identify influential multivariate outliers based on a standard cut-off (4 x mean), and univariate outliers were identified as values deviating from the mean by greater than 3 standard deviations. Outliers were maintained in the dataset and impacts of these outliers on relationships found between tissue mineral concentrations were assessed by removal for a sensitivity analysis. ANOVAs with Tukey’s post-hoc tests were used to evaluate differences in hair trace mineral concentrations based on body location of hair collection (rump versus shoulder). When bivariate normality was not possible, non-parametric tests were used instead (Kruskal-Wallis rank tests and Pairwise-Wilcoxon rank sum tests in place of 1-way ANOVA and Tukey post-hoc tests). In cases where significant differences were noted between the means of element concentrations in samples from different body locations, Spearman rank correlations were reassessed separately for different sample types as a further sensitivity analysis of the initial relationships found based on pooled data. Statistical tests were applied using the following packages: ‘tidyverse’ Version 1.2.1 (2019); ‘dplyr’ Version 0.8.3 (2019); and ‘ggplot2’ Version 3.2.1 (2019). All statistical analyses were done in R Version 3.6.0 [26].

## Results

Age range of caribou sampled with incisors submitted (n = 27) was 3–11 years with median age of 5 years. Of samples collected, 43 livers, 43 kidneys, and hair samples from 31 caribou were sufficient quality for trace mineral analysis (Table 1).

**Table 1:** Trace mineral concentrations determined by ICP-MS (mg/kg, dry weight) in hair (n = 31), liver (n = 43), and kidney (n = 43) samples from wild Northern Mountain caribou via harvest-based sampling in 2016, 2017, and 2018 legal hunting seasons.

	Hair			Liver			Kidney		
	Mean (SD)	Median	Range	Mean (SD)	Median	Range	Mean (SD)	Median	Range
**Cd**	0.03(0.02)*	0.03	0.01–0.08	5.45(4.13)	4.24	1.15–24.10	52.58(28.94)	47.90	13.9–144
**Co**	0.02(0.02)	0.02	0.01–0.11	0.23(0.07)	0.23	0.07–0.38	0.44(0.16)	0.42	0.22–0.95
**Cu**	6.58(0.81)	6.45	5.37–9.00	215.12(128.35)	185.00	3.54–589	24.52(4.74)	24.70	17.8–42.7
**Fe**	17.34(15.69)	12.68	7.84–88.77	470.30(295.61)	401.00	192–1860	192.12(89.55)	179.00	106–503
**Pb**	0.05(0.09)	0.02	0.01–0.43	0.06(0.08)	0.04	0.01–0.52	1.95(12.45)	0.04	0.01–81.60
**Mn**	3.63(6.01)	1.14	0.49–23.07	6.47(3.59)	6.19	0.82–13.7	5.84(2.44)	6.51	1.76–9.80
**Mo**	0.08(0.04)	0.07	0.03–0.18	1.84(0.75)	1.93	0.05–3.65	0.91(0.33)	0.90	0.36–1.84
**Se**	0.36(0.15)	0.35	0.18–0.81	1.70(1.32)	1.38	0.58–7.50	4.92(0.69)	4.91	3.34–6.32
**Zn**	104.96(9.49)	104.48	91.59–137.21	87.21(47.95)	75.20	49–363	127.91(16.14)	124.00	98.1–192.0

* n = 24, because hair samples with concentrations below Limit of Detection were omitted.

**Fig 2:**
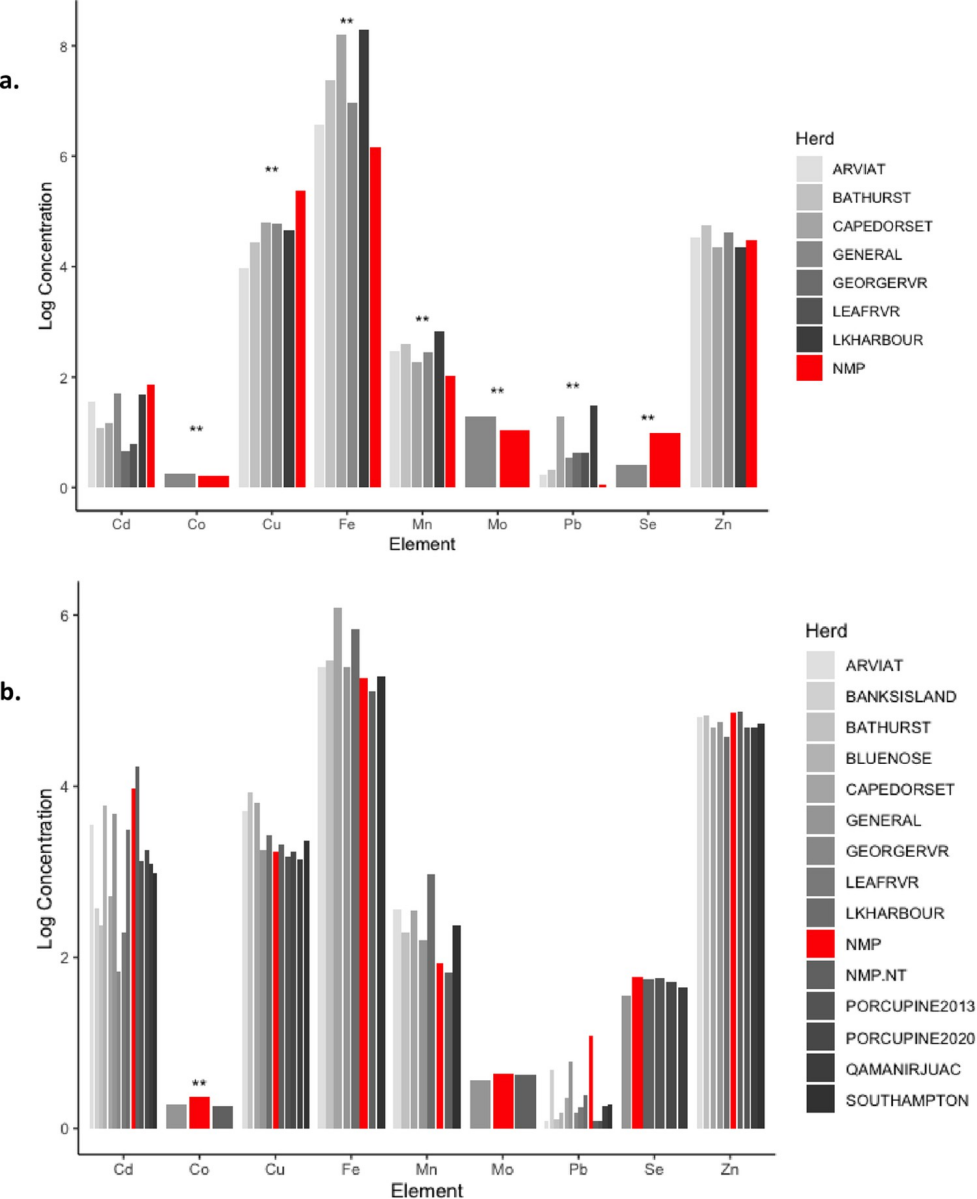
Comparison of trace mineral and heavy metal concentrations (log-transformed; mg/kg, dry weight) found in Northern Mountain caribou (*NMP*; this study) and other caribou herds in various parts of Canada (*Arviat*, *Banks Island*, *Bathurst*, *Bluenose*, *Cape Dorset*, *George River*, *Leave River*, *Lake Harbour*, *Porcupine*, *Qamanijuruac*, *Southampton Herds*; see S1 Table for detailed table and associated references). 2a. Liver mineral concentrations, 2b. kidney mineral concentrations. ** = Northern Mountain caribou mean element concentration (this study) is significantly different than all reported means from other Canadian aribou herds, at p<0.5/*m*, where *m* is the number of ‘other herd’ means being tested for each element (i.e. Bonferroni correction for multiple comparisons).

**Fig 3:**
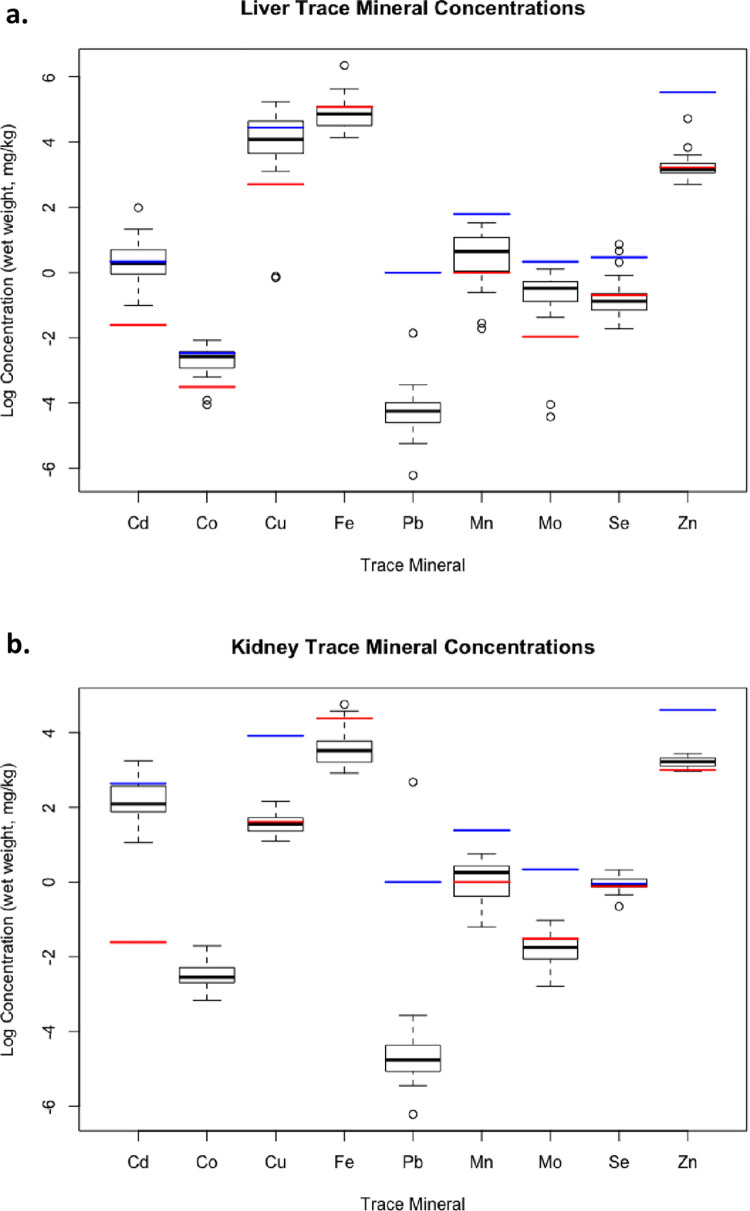
Comparison of mineral values (mg/kg (ppm), wet weight) found in livers (3a.) and kidneys (3b.) of caribou in this study with reference ranges for reindeer and caribou. Box plots visualize medial log mineral concentrations (black solid line) within respective interquartile ranges (IQR), and whiskers identify outliers falling outside IQRs. The red (wide-segmented) and blue (small-segmented) horizontal lines represent the respective lower and upper reference limits for the element, as published in Puls (1994). For some elements, only single thresholds are known or biologically relevant (Fe, Pb; Puls, 1994).

Significant correlations between liver and kidney concentrations were detected for all elements except Co and Cu (Table 2). Mean liver concentrations of Co, Fe, Pb, Mn, and Mo were significantly lower compared to other ecotypes/herds, while liver Cu and Se and renal Co were significantly higher (Fig 2A and 2B). Liver and/or kidney mineral concentrations in a high proportion of animals fell outside of the reference ranges for captive or semi-domesticated reindeer and caribou (*Rangifer spp*.) [27] (Fig 3A and 3B). These included: 55.8% renal Cu, 74.4% hepatic and 95.3% renal Fe, 25.6% hepatic and 39.5% renal Mn, 76.7% renal Mo, 65.1% hepatic and 37.2% renal Se, and 53.5% hepatic Zn below the reference ranges and 48.8% hepatic and 25.6% renal Cd, 30.2% hepatic Co, 32.6% hepatic Cu, and 39.5% renal Se above reference ranges.

**Table 2:** Spearman Rank correlation coefficients (rho) for liver and kidney trace mineral/heavy metal concentrations from same individuals (n = 34) of free-ranging Northern Mountain caribou.

Element	Rho	P-VALUE
**Cd**	0.78**	0.00
**Co**	-0.04	0.81
**Cu**	0.06	0.75
**Fe**	0.39**	0.02
**Pb**	0.60**	0.00
**Mn**	0.80**	0.00
**Mo**	0.66**	0.00
**Se**	0.45**	0.01
**Zn**	0.42**	0.01

* = significant at p ≤ 0.10

** = significant at p ≤ 0.05

Element concentrations detected in hair fell below the method LOQ for Co (90.6% samples), Mo (25% samples), Pb (100% samples), and Cd (100% samples). For Cd, 22.6% of samples (n = 31) fell below detectable limits and were non-quantifiable (Table 1).

**Fig 4:**
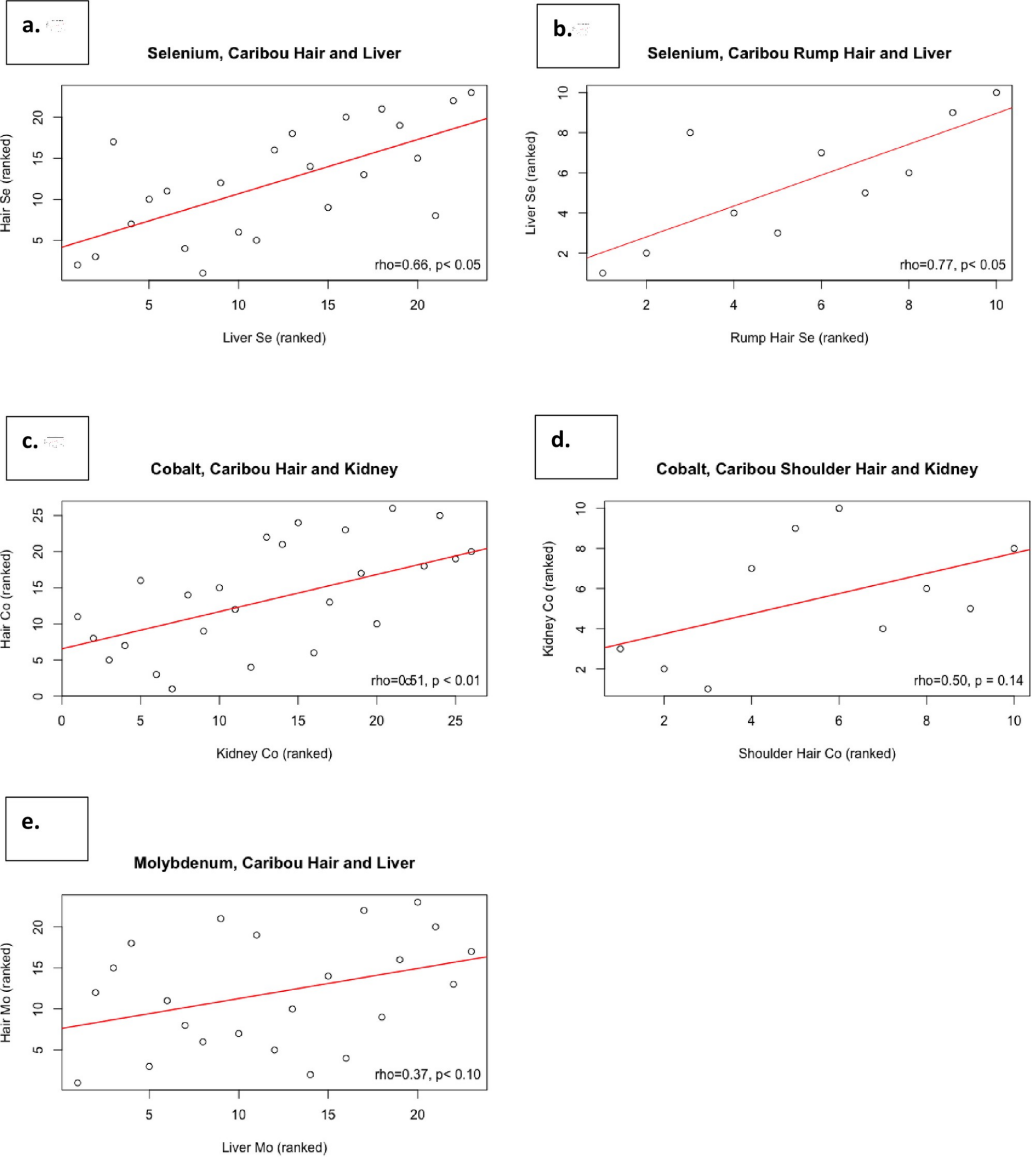
a. Plot of rank correlations between kidney and hair concentrations of selenium (Se) in same individuals (n = 23) of Northern Mountain caribou. Legend indicates Spearman rank coefficient (rho) and p-value. b. Plot of rank correlations between liver and shoulder hair concentrations of selenium (Se) in same individuals (n = 10). Legend indicates Spearman rank coefficient (rho) and p-value. c. Plot of rank correlations between kidney and hair concentrations of cobalt (Co) in same individuals (n = 23) of Northern Mountain caribou. Legend indicates Spearman rank coefficient (rho) and p-value. d. Plot of rank correlations between kidney and shoulder hair concentrations of cobalt (Co) in same individuals (n = 10). Legend indicates Spearman rank coefficient (rho) and p-value. e. Plot of rank correlations between liver and hair concentrations of molybdenum (Mo) in same individuals (n = 23) of Northern Mountain caribou. Legend indicates Spearman rank coefficient (rho) and p-value.

There was a significant strong positive monotonic correlation between liver and hair Se concentrations (Fig 4A; rho = 0.66, p = 0.001), and a significant moderate positive rank correlation between kidney and hair Co (Fig 4C; rho = 0.51, p = 0.008), and a moderate positive rank correlation between liver and hair Mo concentrations (Fig 4E; rho = 0.37, p = 0.085). There were no other significant correlations found between concentrations in hair and liver or kidney for the remaining elements (Table 3; rho < 0.35 and p > 0.05).

**Table 3:** Spearman Rank correlation coefficients (rho) for correlations between hair and liver (n = 23) and hair and kidney (n = 26) trace mineral concentrations from same individuals of free-ranging Northern Mountain caribou.

Element	Liver		Kidney	
Hair	rho	p-value	rho	p-value
CD	0.31	0.15	0.28	0.16
CO	0.16	0.46	0.51**	0.01
CU	-0.03	0.89	0.24	0.24
FE	-0.21	0.35	-0.08	0.71
PB	-0.07	0.76	0.10	0.64
MN	0.01	0.97	-0.05	0.81
MO	0.37*	0.08	0.12	0.57
SE	0.66**	0.00	0.31	0.13
ZN	0.07	0.77	0.26	0.20

* = significant at p ≤ 0.10

** = significant at p ≤ 0.05

With influential outliers removed, the relationships between liver and hair concentrations of Se and Mo remained the same (rho = 0.60, 0.39 and p = 0.005, 0.073, respectively), as did the relationship between kidney and hair concentrations of Co (rho = 0.54, p = 0.008); no new correlations were detected.

We assessed for differences between mean element concentrations in hair from the shoulder region or rump hide. Se and Co concentrations were significantly influenced by site of hair collection (p = 0.02 and p = 0.01, respectively). For Co, the relationships between hair and kidney mineral concentrations remained in a positive direction despite factoring in body location of sampling but were not significant (Fig 4B: rho_shoulder_ = 0.50, p_shoulder_ = 0.14; rho_rump_ = 0.13, p_rump_ = 0.70). Spearman rank coefficients (rho) were 0.40 (p_shoulder_ = 0.29) and 0.77 (p_rump_ = 0.01; Fig 4B) for the relationships between liver Se and Se in plucked shoulder hairs and shaved rump hairs, respectively.

## Discussion

We have established baselines of trace mineral and heavy metal status in the liver, kidney, and hair of Northern Mountain caribou harvested in the late summer/fall, and also have assessed associations between hair and organ trace mineral levels. The consistent and intentional age, sex, and seasonal bias in sampling (adult, males, fall hunting season) accounted for several known spatial, temporal, demographic, and ecological confounding factors. This resulted in development of robust reference values and a basis for future comparisons for this caribou demographic. This sample design also provided the ideal timing for comparing organ versus hair trace minerals at the end of the growing season, as opposed to later in the year when organ stores could become depleted [5].

Sampled caribou had no obvious signs of mineral deficiency or toxicity, however, concentrations of several trace minerals (Co, Cu, Fe, Mn, Mo, Pb, and Se in livers and Co in kidneys) were significantly different from those of other Canadian caribou herds (Fig 2A and 2B) and/or fell outside the ‘normal’ ranges published for *Rangifer sp*. (domestic reindeer and caribou; [27]) (see Fig 3A and 3B). Differences among free-ranging herds may be attributed to differing exposures or physiologic tolerances based on ecotype, forage preference, local geology and soil mineral levels, sex, age, season, and habitat selection [28, 29]. Differences from the ‘normal’ ranges provided by [27] may be explained in part by the fact that captive animals have a supplemented and much more consistent diet compared to the wild *Rangifer*. Differences in analytical methods and reporting (e.g., ranges from Puls [27] are provided in wet weight (ppm)) may also account for variance among populations. These discrepancies highlight the needs for standardized methods and updated trace mineral reference ranges that take into account animal age, sex, geography, and ecotype.

For all elements except Co and Cu, concentrations found in paired liver and kidney tissues were significantly positively correlated (p <0.05; Table 2). Correlations between liver and kidney concentrations for some elements were similarly noted in Svalbard reindeer [30].

### Hair trace minerals and correlations with organ mineral concentrations

Caribou hair is formed in a defined time period before becoming separated from bodily metabolic activity [13], so hair shafts should reflect the animal’s metabolic state during time of hair formation. Trace minerals are cumulatively incorporated into growing hair from circulating blood and would predictably be most concentrated late in the phase of active hair synthesis [13]. The caribou hair growth period begins late spring/early summer and continues until the late fall [31–33]. Mature bulls generally moult the earliest and have fully developed new coats by late summer/onset of rut [31, 32]. Our samples were, therefore, ideally representative of mineral deposition in hair between the late spring and time of sampling (fall), and uniquely positioned our study to better control for variability introduced by confounding factors like season, diet, life stage, and sex [14, 28, 34]. Findings in moose support this hypothesis, describing hair mineral concentrations as most accurately representative of dietary mineral intake in the late summer and fall [1, 35]. We would expect that the three sample types assessed for mineral concentrations in this study would be representative of a common time period and the associated seasonal diet. Organ levels might fluctuate more, however, as they also reflect historic intake (deficiencies or excesses) and storage for some elements, and are responsive to acute dietary changes and metabolic demands. In contrast, hair levels reflect a cumulative deposition over the summer and levels cannot be ‘depleted’ like organ levels can.

We observed correlations between hair mineral levels and liver and/or kidney levels for some elements (Se, Co, and Mo) but not others; these differences could be attributed to a number of factors. Some trace minerals are tightly hemostatically regulated to stay within specific circulating physiologic ranges, with excess incorporated into organs for storage or excreted. We would expect the mineral incorporation into the hair to be consistent with levels in the circulating blood regardless of liver and kidney stores, unless the animal was sufficiently deficient to result in sustained reduced circulating concentrations. We had no indications of significant deficiencies in our sampled animals, perhaps explaining the absence of correlations for many elements.

Additionally, preference, palatability, and availability of different forage species to caribou, as well as the mineral status and bioavailability of those plants, varies seasonally and spatially [9]. Shorter term exposure to a trace element may be reflected in liver or kidney concentrations, but perhaps not sensitive in hair if exposure was not maintained chronically. Further, complex, variable metabolic interactions exist among some trace minerals, and different minerals and their concentrations can thus influence the absorption, bioavailability, and organ storage activities of each other [13, 36, 37], perhaps complicating identification of relationships between dietary intake, organ levels, and hair concentrations.

#### Selenium, cobalt, and molybdenum

Correlations between hair and organ levels were detected for three key elements: Se, Co, and Mo. Selenium plays an essential role in ruminants supporting normal immune function, and imbalances can cause clinical disease, reproductive issues, and even death [3] (see Table 4). We observed a strong positive association between hair and liver Se levels (Fig 4). These results are consistent with those in other ruminant species where hair Se has been correlated with dietary intake [34, 38] and is strongly associated with Se levels in liver and whole blood [14 (free-ranging mule deer), 39 (domestic cattle)]. Hair Se above and below specific thresholds can be diagnostic of clinical Se toxicity or deficiency in cattle [38, 40]. Our findings suggest hair is a good indicator of Se status in mountain caribou, and therefore is a suitable, non-invasive, and easily acquired tool that can inform an important indicator (Se status) of caribou nutrition, immune function, reproductive potential, and population success [3] (Mosbacher et *al*., unpublished; see Table 4).

**Table 4:** Heavy metals and trace minerals assessed in this study along with brief explanations of their functional relevance and/or the clinical effects of toxicities and/or deficiencies expected or previously observed in cervids or ruminants.

MINERAL	SIGNS OF NUTRITIONAL IMBALANCE/TOXICITY
**HEAVY METALS (CADMIUM (CD) AND LEAD (PB))**	• Widely distributed volatile heavy metals, exist both naturally in the environment and also from anthropogenic sources of environmental contamination • Elevations in tissue Cd and Pb in wild ungulates been associated with point source contamination from proximity to industrial activity, and long-range transport in air masses [30, 41] • Dietary exposure primary route of contaminant exposure in most free-ranging mammals [34, 41] • At elevated levels, cause hepatic and renal damage, bone deterioration, neurological disease, and increased mortality [42–44].
**COBALT (CO)**	• Core component of vitamin B12 • Deficiency of Co, (and thus vitamin B12) can lead to macrocytic anaemia. Overload can reduce thyroid function in mammals [45, 46] • Deficiency in ruminants is linked to poor condition, weakness, and delayed or poor reproductive capacity [27]. Frank et *al*. [47] attributed ‘moose sickness’ in Eastern Canada to Co/Vitamin B12 deficiency, presenting with progressive wasting and generalized neuropathy
**COPPER (CU)**	• Deficiency in cervids associated with infertility, hoof and antler abnormalities, poor condition, weakness, impaired growth, inappropriate keratinization, and neurologic disease [27] • Deficiency in domestic ruminants associated with impaired immune function, increased susceptibilities to infectious disease [6] • Linked deficiency to population declines and poor reproductive rates in moose [4]
**IRON (FE)**	• Deficiency in ruminants associated with anemia, can cause impaired growth and reduced calf immune capacity • High Fe can cause deficiencies in other elements (including Cd, Co, Cu, Mn, Se, and Zn), and those elements can reduce absorption or bioavailability of Fe [27]
**MANGANESE (MN)**	• Deficiency in ruminants associated with decreased reproductive potential in adults and malformation of bones and joints, low birth weight, and paralysis in calves [27, 38]
**MOLYBDENUM (MO)**	• Toxicity in ruminants delays reproductive age, decrease reproductive success, and potentially influences calf growth rates [27] • Mo negatively influences efficiency of Cu absorption in ruminants, and toxic levels can trigger and be aggravated by Cu deficiency [8, 27, 48]
**SELENIUM (SE)**	• Severe deficiencies manifest in syndromes like White Muscle Disease (WMD), typically in young ruminants and associated with delayed growth, unthriftiness, muscle stiffness, respiratory distress, and even sudden death [3] • Affected individuals possibly more susceptible to predation, compounded by early weaning by Se-deficient cows with low milk production [49] • Essential for normal immune function; deficiency can influence an animal’s resistance to infections [50] • In subclinical Se deficiency in ruminants, reproductive success in males and females is affected and includes neonatal mortality, poor birth weight, poor conception rates, impaired male fertility, and poor success of offspring [3]

Molybdenum imbalances can impact reproduction and growth in ruminants [8, 27, 48] (see Table 4). We detected a significant moderate association between liver and hair Mo levels. Though quantifiable amounts of Mo were detected for all hair samples analyzed, our degree of certainty/precision was decreased for the 25% of samples with concentrations below method LOQ. This limitation might explain extra variability in our model. Cunningham and Hogan showed that increasing dietary Mo intake induced elevated Mo levels in hair of domestic cattle, and also caused hair Cu levels to decline [48]. Mo toxicity in cattle is indicated specifically when higher levels are associated with low Cu in circulation; even when serum Cu is adequately managed by hemostatic mechanisms, excess circulating Mo may influence biological availability of Cu and can even be exacerbated by Cu deficiency [27]. Though further exploration is warranted, our findings might support the monitoring of dietary Mo status, and potentially informing Cu dynamics, in mountain caribou using body hair samples.

Cobalt imbalances in ruminants can be associated with anemia, altered metabolism, weakness, and poor reproductive health [27] (see Table 4). We found a significant moderate correlation between the concentrations of Co in hair and kidney (p = 0.01), but not with hair and liver. Liver is the primary storage organ best representative of Co and vitamin B12 levels [45, 47, 51]. Hair can be a good indicator of Co status in horses, with the relationship between dietary Co intake and hair Co concentrations more apparent at higher concentrations [52]. In our study, 93.5% of samples had Co concentrations below the LOQ, which introduced greater variability when assessing correlations. Mean hair Co concentrations also differed significantly based on location of sampling, and hair Co and kidney Co were no longer significantly related when relationships were assessed for each location of sampling separately–this could be in part associated with loss of statistical power due to an already limited sample size. While hair Co has shown some promise in previous studies as an indicator of health in other species, our results remain inconclusive likely due to the low concentrations of Co accumulated in hair samples. Increasing the mass of hair analyzed to reach levels above method LOQs–although not possible in this study because of a limited volume of hair available from opportunistic sampling–will likely decrease variability in results and improve our understanding of the relationship between hair and organ Co.

#### Other minerals

We did not detect significant associations between organ and hair levels of the key trace elements Cu, Mn, Fe, or Zn.

Copper is an essential element and deficiencies are associated with weakness, neurological symptoms, impaired immunity and reproductive capacity, among other clinical syndromes [6, 27] (see Table 4). Low hair Cu concentrations have been linked to clinical signs of deficiency and low dietary Cu intake in moose and red deer [4, 53]. Our absence of an association may be linked to the natural dynamics of Cu in the body. Serum Cu concentrations typically do not reflect liver Cu status until substantial depletion of Cu stores because of tight homeostatic regulation [27]. Concentrations of Cu incorporated into the hair shaft are not expected to reflect dietary intake or Cu stores when liver concentrations are above a threshold of 20 ppm wet weight [27, 54]. Hair is likely a better indicator of Cu status when a caribou is Cu-deficient, but we could not explore this with our sample set as 41/43 (95.3%) of submitted livers had Cu concentrations above the 20 ppm threshold. Cu-status is an important measure of caribou health [15] (see Table 4), and hair Cu concentration may be an effective indicator for identifying Cu-deficiencies.

Similar to Cu, manganese is a mineral under tight regulatory control, maintained at stable tissue and blood concentrations, unless deficient and below some threshold [55]. Colour of hair and external exposure to/contamination by grasses are confounding factors that influenced hair Mn concentrations in domestic ruminants [38]. Mountain caribou, particularly in the summer and fall seasons, have coats that vary considerably in hair colour, which may explain variability in hair Mn levels.

Previous studies have shown that hair is not a useful index of Fe status in ruminants [13, 14], and that Zn levels in ruminant hair tend to be highly variable and not reflective of the severity of Zn deficiencies or predictive of clinical signs associated with imbalances [34, 56]. The metabolic interactions among elements such as Fe, Zn, Mo, and Cu, and their interdependent fluctuations in serum and organ levels and bioavailability, may complicate the predictability and consistency of deposition of these minerals in hair in relation to dietary intake or metabolic status [13, 36, 37]. Interpretation of hair mineral concentrations and their relationships with dietary or organ levels may be more complicated and dynamic in these cases.

#### Heavy metals

Heavy metal contaminants can accumulate in wild ruminants like caribou and have potential negative impacts on animal health, reproduction, and survival. In the case of caribou, human consumption of meat and organs can also introduce potential public health concerns [5]. The hair concentrations of the heavy metals Cd and Pb in our study were very low, all falling below method LOQ. This precluded true interpretation of correlations between hair and organs. Ruminant hair is considered a poor indicator of dietary Cd intake compared to tissues since minimal, even negligible, amounts are deposited in hair compared to kidney or liver (0.0165% of single oral dose radioactive-Cd [44, 56]). Lead in hair also tends to correlate poorly with serum or liver content, except in cases of primarily exogenous lead contamination of hair or chronic poisoning [34]. A higher mass of hair analysed for heavy metal concentration may improve detectability and confidence in levels of Cd and Pb. Hair is a commonly used media to assess for heavy metal toxicity in humans [57] and could conceivably be as useful in free-ranging wildlife. Though these metal concentrations were very low in our sample set, our study provides a point of comparison; detecting values in caribou hair/tissues well above LOQ may be a meaningful indication of excess exposure to toxic metals.

## Conclusions

The wildlife conservation and management communities are increasingly recognizing the importance of more holistic assessments of wildlife health [58, 59], including caribou health [15, 18]. Trace mineral imbalances can result in impaired immune function, reduced productivity, and changing population demographics, but are infrequently assessed when monitoring population health and status [1, 3–5, 7, 8, Mosbacher et al., unpublished]. In the face of the financial and logistical challenges to monitoring remote wildlife populations and Species at Risk, creative solutions to assessing individual and population health are needed. Caribou hair is a non-invasive, easily collected, and simply stored biological sample that can be gathered by harvesters, outfitters, anyone with direct access to animals, or even passively from the environment once moulted [34, 38, Mosbacher et *al*., unpublished]. We have demonstrated hair as a good health monitoring tool for the assessment of Se, Co, and Mo status in caribou, suspect it would be effective for detecting deficiencies in Cu and Mn, and potentially could serve as an indicator of certain heavy metal toxicities.

Mineral imbalances can have cascading health impacts. Incorporating the assessment of hair mineral profiles into harvest-based sampling and other surveillance activities is ultimately a practical option that generates critical information about wildlife health. Such information can then be used to inform conservation and range management planning.

## Supporting information

S1 Table Comparison of trace mineral concentrations (mg.kg, dry weight) found in Northern Mountain caribou (this study) and other caribou herds/ecotypes in various parts of Canada. * = Northern Mountain caribou mean element concentration (this study) is significantly different than all available means from other Canadian herds, at p < 0.05/*m*, where *m* is the number of ‘other herd’ means being tested against for each element (i.e. Bonferroni correction applied due to multiple comparisons).
(PDF)

S1 Graphical abstract (PDF)

## References

[1] O’Hara TM, Carroll G, Barboza P, Mueller K, Blake J, Woshner V, et al. Mineral and heavy metal status as related to a mortality event and poor recruitment in a moose population in Alaska. J Wildl Dis. 2001; 37(3):509–522. doi: 10.7589/0090-3558-37.3.509 11504224

[2] Weiss G and Carver P. Role of divalent metals for infectious diseases susceptibility and outcome. Clin Microbiol Inf. 2017; 24(1):16–23. 10.1016/j.cmi.2017.01.018 28143784

[3] Flueck WT, Smith-Flueck JM, Mionczynski J, and Mincher BJ. The implications of selenium deficiency for wild herbivore conservation: a review. Eur J Wildl Res. 2012. 58:761–780.

[4] Flynn A, Franzmann AW, Arneson PD, and Oldemeyer JL. Indications of copper deficiency in a subpopulation of Alaskan moose. J Nutr. 1977; 107(7):1182–1189. doi: 10.1093/jn/107.7.1182 874562

[5] Gamberg M, Cuyler C, and Wang X. Contaminants in two West Greenland caribou populations. Sci Total Environ. 2016; 554–555:329–336. doi: 10.1016/j.scitotenv.2016.02.154 26956180

[6] Scaletti RW, Trammell DS, Smith BA, and Harmon RJ. Role of dietary copper in enhancing resistance to *Escherichia coli* mastitis. J Dairy Sci. 2003; 86(4):1240–1249. doi: 10.3168/jds.S0022-0302(03)73708-4 12741549

[7] Flueck WT. Effect of trace elements on population dynamics: Selenium deficiency in free-ranging black-tailed deer. Ecology. 1994; 75(3): 807–812.

[8] Pollock B and Roger E. Trace elements status of moose and white-tailed deer in Nova Scotia. Alces. 2007; 43:61–77.

[9] Oster KW, Barboza PS, Gustine DD, Joly K, and Shivley RD. Mineral constraints on arctic caribou (Rangifer tarandus): a spatial and phenological perspective. Ecosphere. 2018; 9(3): e02160.

[10] Durkalec M, Nawrocka A, Krysiak M, Larska M, Kmiecik M, and Posyniak A. Trace elements in the liver of captive and free-ranging European bison (*Bison bonasus L*.). Chemosphere. 2018; 193:454–463. doi: 10.1016/j.chemosphere.2017.11.050 29154121

[11] Blakely BR, Kutz SJ, Tedesco SC, Flood PF. Trace mineral and vitamin concentrations in the liver and serum of wild muskoxen from Victoria Island. J Wildl Dis. 2000; 36(2):301–307. doi: 10.7589/0090-3558-36.2.301 10813612

[12] Vermunt JJ and West DM. Predicting copper status in beef cattle using serum copper concentrations. N Z Vet J. 1994; 42:194–195. doi: 10.1080/00480169.1994.35821 16031781

[13] Combs DK. Hair analysis as an indicator of mineral status in livestock. J Anim Sci. 1987; 65:1753–1758. doi: 10.2527/jas1987.6561753x 3327852

[14] Roug A, Swift PK, Gerstenberg G, Woods LW, Kreuder-Johnson C, Torres SG, et al. Comparison of trace mineral concentrations in tail hair, body hair, blood, and liver of mule deer (*Odocoileus hemionus*) in California. J Vet Diagn Invest. 2015; 27(3): 295–305. doi: 10.1177/1040638715577826 25862714

[15] Bondo KJ, Macbeth B, Schwantje H, Orsel K, Culling D, Culling B, et al. Health survey of boreal caribou (*Rangifer tarandus caribou*) in northeastern British Columbia, Canada. J Wildl Dis. 2018; 55(3):544–562. 10.7589/2018-01-018 30605390

[16] Di Francesco J, Navarro-Gonzalez N, Wynne-Edwards K, Peacock S, Leclerc LM, Tomaselli M, et al. Qiviut cortisol in muskoxen as a potential tool for informing conservation strategies. Conserv Physiol. 2017; 5(1), cox052. doi: 10.1093/conphys/cox052 28948023 PMC5601961

[17] Koren L, Bryan H, Matas D, Tinman S, Fahlman Å, Whiteside D, Smits J, et al. Towards the validation of endogenous steroid testing in wildlife hair. J Appl Ecol. 2019; 56: 547–561.

[18] Kutz S, Ducrocq J, Cuyler C, Elkin B, Gunn A, Kolpashikov L, et al. Standardized monitoring of *Rangifer* health during International Polar Year. Rangifer (Special Issue). 2013; 33:91–114.

[19] Hegel TM and Russell K. Status of northern mountain caribou (*Rangifer tarandus caribou*) in Yukon, Canada. Rangifer (Special Issue). 2011; 33(21):59–70.

[20] COSEWIC. COSEWIC assessment and status report on the caribou *Rangifer tarandus*, Northern Mountain population, Central Mountain population and Southern Mountain population in Canada. Committee on the Status of Endangered Wildlife in Canada, Ottawa, Ontario. 2014. Xxii + 113 pp.

[21] Environment Canada. Management Plan for the Northern Mountain Population of Woodland Caribou (*Rangifer tarandus caribou*) in Canada. *Species at Risk Act* Management Plan Series. Environment Canada, Ottawa. 2012. vii + 79 pp. Available from https://www.sararegistry.gc.ca/virtual_sara/files/plans/mp_woodland_caribou_northern_mountain_population_e.pdf. [accessed September 2020].

[22] Matson G.M. Workbook for Cementum Analysis. Milltown: Matson’s Laboratory; 1981.

[23] Vikøren T, Kristoffersen AB, Lierhagen S, and Handeland K. A comparative study of hepatic trace element levels in wild moose, roe deer, and reindeer from Norway. J Wildl Dis. 2011; 47(3): 661–672. doi: 10.7589/0090-3558-47.3.661 21719831

[24] Horvath S. British Columbia Environmental Laboratory Manual. Water and Air Monitoring and Reporting; Water, Air and Climate Change Branch. Victoria: Ministry of Environment; 2005;

[25] EPA. 1998. "Method 6020A (SW-846): Inductively Coupled Plasma-Mass Spectrometry," Revision 1.

[26] R Core Team. R: A Language and Environment for Statistical Computing. R Found. Stat. Comput. Vienna, Austria. 2019. Available online: https://www.R-project.org/

[27] Puls R. Mineral levels in animal health: Diagnostic data. 1994. Sherpa International, Clearbrook, British Columbia. pp. 238.

[28] Gamberg M, Pratte I, Brammer J, Cuyler C, Elkin B, Gurney K, et al. Renal trace elements in barren-ground caribou subpopulations: Temporal trends and differing effects of sex, age, and season. Sci Total Environ. 2020; 724:138305. doi: 10.1016/j.scitotenv.2020.138305 32272411

[29] Larter NC and Nagy JA. A comparison of heavy metal levels in the kidneys of High Arctic and mainland caribou populations in the Northwest Territories of Canada. Sci Total Environ. 2000; 246:109–119. doi: 10.1016/s0048-9697(99)00418-0 10696717

[30] Borch-Iohnsen B, Nilssen KJ, and Norheim G. Influence of season and diet on liver and kidney content of essential elements and heavy metals in Svalbard reindeer. Biol Trace Elem Res. 1996; 51:235–247. doi: 10.1007/BF02784078 8727671

[31] Cuyler C and Øritsland NA. Do seasonal changes in Svalbard reindeer fur have relevance for heat transfer? Rangifer. 2002; 22(2):133–142.

[32] Drucker DG, Hobson KA, Ouellet JP, and Courtois R. Influence of forage preferences and habitat use on 13C and 15N abundance in wild caribou (*Rangifer tarandus caribou*) and moose (*Alces alces*) from Canada. Isot Environ Healt S. 2010; 46(1):107–121. 10.1080/10256010903388410 20229388

[33] Pedersen AO. 2019. *Rangifer* Biology and Adaptations. In: Tryland M. and Kutz S.J., editors. Reindeer and caribou health and disease. Boca Raton: Taylor and Francis Group; 2005. pp. 54–67.

[34] Combs DK, Goodrich RD, and Meiske JC. Mineral concentrations in hair as indicators of mineral status: A review. J Anim Sci. 1982; 54:391–398. doi: 10.2527/jas1982.542391x 7042673

[35] Bubenik AB. Evolution, Taxonomy and Morphophysiology. In: Franzmann AW and Schwartz CC, editors. Ecology and Management of North American Moose. Washington: Wildlife Management Institute; 1997; pp. 77–123.

[36] Collins JF, Prohaska JR, and Knutson M. Metabolic crossroads of iron and copper. Nutr Rev. 2010; 68(3):133–147. doi: 10.1111/j.1753-4887.2010.00271.x 20384844 PMC3690345

[37] Counotte G, Holzhauer M, Carp-van Dijken S, Muskens J, and Van der Merwe D. Levels of trace elements and potential toxic elements in bovine livers: A trend analysis from 2007 to 2018. PLoS ONE. 2019; 14(4):e0214584. doi: 10.1371/journal.pone.0214584 30964882 PMC6456170

[38] Hidiroglou M, Carson RB, and Brossard GA. Influence of selenium on the selenium contents of hair and the incidence of nutritional muscular disease in beef cattle. Can J Anim Sci. 1965; 45:197.

[39] Christodoulopoulos G, Roubies N, Karatzias H, and Papasteriadis A. Selenium concentration in blood and hair of Holstein dairy cows. Biol Trace Elem. Res. 2003; 91:145–150. doi: 10.1385/BTER:91:2:145 12719609

[40] Olson OE. Selenium and a toxic factor in animal nutrition. In: Proceedings of Georgia Nutritional Conference. Athens: Univ of Georgia; 1969; p. 68.

[41] Gamberg M and Scheuhammer AM. Cadmium in caribou and muskoxen from the Canadian Yukon and Northwest Territories. Sci Total Environ. 1994; 143:221–234. doi: 10.1016/0048-9697(94)90459-6 7911600

[42] Burger J. Assessment and management of risk to wildlife from cadmium. Sci Total Environ. 2008; 389:37–45. doi: 10.1016/j.scitotenv.2007.08.037 17910979

[43] Hamilton DL and Smith MW. Inhibition of intestinal calcium uptake by cadmium and the effect of a low calcium diet on cadmium retention. Environ Res. 1978; 15(2):175–184. doi: 10.1016/0013-9351(78)90094-4 668651

[44] Neathery MW and Miller WJ. Metabolism and toxicity of cadmium, mercury, and lead in animals: A review. J Dairy Sci. 1975; 58(12):1767–1781. doi: 10.3168/jds.S0022-0302(75)84785-0 1107364

[45] Hassan AA, Rylander C, Sandanger TM, and Brustad M. Copper, cobalt, chromium in meat, liver, tallow, and bone marrow from semi-domesticated reindeer (*Rangifer tarandus tarandus L*.) in northern Norway. Food Publ Health. 2013; 3(3):154–160.

[46] McDowell LR. Minerals in animal and human nutrition, 2^nd^ Edition. Elsevier Science BV, Amsterdam, Netherlands. 2003. 644 pp.

[47] Frank A, McPartlin J, and Danielsson R. Nova Scotia moose mystery–a moose sickness related to cobalt and vitamin B_12_ deficiency. Sci Total Environ. 2004; 318:89–100. doi: 10.1016/S0048-9697(03)00374-7 14654277

[48] Cunningham IJ and Hogan KG. The influence of diet on the copper and molybdenum contents of hair, hoof, and wool. New Zeal J Agri Res. 1958; 1(6): 841–846.

[49] Hnilicka PA, Mionczynski J, Mincher BJ, States J, Hinschberger M, Oberlie S, et al. Bighorn sheep lamb survival, trace minerals, rainfall, and air pollution: are there any connections? Bienn Symp, North Wild Sheep and Goat Council. 2004; 13: 69–94.

[50] Kasaikina MV, Kravtsova MA, Lee BC, Seravalli J, Peterson DA, Walter J, et al. Dietary selenium affects host selenoproteome expression by influencing the gut microbiota. FASEB J. 2011; 25(7):2492–2499. doi: 10.1096/fj.11-181990 21493887 PMC3114522

[51] Andrews ED, Hart LI, and Stephenson BJ. Vitamin B12 and cobalt in livers from grazing cobalt-deficient lambs and from others given various cobalt supplements. New Zeal J Agr Res. 1960; 3(2):364–376.

[52] Ghorbani A, Mohit A, and Kuhi HD. Effects of dietary mineral intake on hair and serum mineral contents of horses. J Equine Vet Sci. 2015; 35:295–300.

[53] Tolgyesi G and Bencze L. The microelement contents of plants consumed by wild mammals, particularly by big game in different game management regions. In: Mills CF, Livingstone S, editors. Trace Element Metabolism in Animals. 1970. pp. 416–418.

[54] Kellaway RC, Sitorus P, and Leibholz M. The use of copper levels in hair to diagnose hypocuprosis. Res Vet Sci. 1978; 24:352–357. 674847

[55] Aschner JL and Aschner M. Nutritional aspects of manganese homeostasis. Mol Aspects Med. 2005; 26(4–5): 353–362. doi: 10.1016/j.mam.2005.07.003 16099026 PMC6309959

[56] Miller WJ. Zinc nutrition of cattle: A review. J Dairy Sci. 1978; 53(8):1123–1135. 10.3168/jds.S0022-0302(70)86355-X 4918951

[57] Michalak I, Wolowiec P, and Chojnacka K. Determination of exposure to lead of subjects from southwestern Poland by human hair analysis. Environ Monit Assess. 2014; 186(4): 2259–2267. doi: 10.1007/s10661-013-3534-3 24346348 PMC3939012

[58] Stephen C. Toward a Modernized Definition of Wildlife Health. J Wildl Dis. 2014; 50(3): 427–430. doi: 10.7589/2013-11-305 24807179

[59] Wittrock J, Duncan C, and Stephen C. A determinants of health conceptual model for fish and wildlife health. J. Wildl. Dis. 2019; 55(2): 285–297. doi: 10.7589/2018-05-118 30289339

