# Supplementary material for: Evaluating the use of hair as a non-invasive indicator of trace mineral status in woodland caribou (Rangifer tarandus caribou)
Source: PLoS One. 2022 Jun 28;17(6):e0269441. doi: 10.1371/journal.pone.0269441 (PMC9239472; doi:10.1371/journal.pone.0269441)
Supplement: S1 Table — * = Northern Mountain caribou mean element concentration (this study) is significantly different than all available means from other Canadian herds, at p < 0.05/m, where m is the number of ‘other herd’ means being tested against for each element (i.e. Bonferroni correction applied due to multiple comparisons). (PDF) [file pone.0269441.s001.pdf]

Supplementary Table. Comparison of trace mineral concentrations (mg/kg, dry weight) found in Northern Mountain caribou (this study) and other caribou herds/ecotypes in various parts of Canada.

	Liver			Kidney		
Element	NM Caribou (mean (SD))	Other Herds (mean (herd, *reference*))	Comparison (t-test)	NM Caribou (mean (SD))	Other Herds (mean (herd, *reference*))	Comparison (t-test)
Cd	5.45(4.13)	1.96 (Bathurst, *Elkin and Bethke, 1995*)<br>3.69 (Arviat, *Elkin and Bethke, 1995*)<br>2.24 (Cape Dorset, *Elkin and Bethke, 1995*)<br>4.39 (Lake Harbour, *Elkin and Bethke, 1995*)<br>1.18 (Leaf River, *Robillard et al.,2002*)<br>0.94 (George River, *Robillard et al., 2002*)<br>4.52 (General, *Gamberg (unpublished data)*)	NM Higher	52.58(28.94)	9.68 (Bathurst, *Elkin and Bethke, 1995*)<br>33.87 (Arviat, *Elkin and Bethke, 1995*)<br>18.79 (Southampton, *Elkin and Bethke, 1995*)<br>14.06 (Cape Dorset, *Elkin and Bethke, 1995*)<br>31.98 (Lake Harbour, *Elkin and Bethke, 1995*)<br>8.93 (Leaf River, *Robillard et al.,2002*)<br>5.23 (George River, *Robillard et al., 2002*)<br>12.23 (Banks Island, *Larter and Nagy, 2000*)<br>42.60 (Bluenose, *Larter and Nagy, 2000*)<br>21.90 (Porcupine, *Gamberg, 2013*)<br>21.00 (Qamanirjuac, *Gamberg, 2013*)<br>38.8 (General, *Gamberg (unpublished data)*)<br>24.8 (Porcupine, *Gamberg et al., 2020*)	NM Higher
Co	0.23(0.07)	0.30 (General, *Gamberg (unpublished data*)	NM Lower*	0.44(0.16)	0.32 (General, *Gamberg (unpublished data)*)	NM Higher*
Cu	215.12(128.35)	83.63 (Bathurst, *Elkin and Bethke, 1995*)<br>51.85 (Arviat, *Elkin and Bethke, 1995*)<br>120.76 (Cape Dorset, *Elkin and Bethke, 1995*)<br>105.36 (Lake Harbour, *Elkin and Bethke, 1995*)	NM Higher*	24.52(4.74)	49.73 (Bathurst, *Elkin and Bethke, 1995*)<br>40.1 (Arviat, *Elkin and Bethke, 1995*)<br>27.76 (Southampton, *Elkin and Bethke, 1995*)<br>43.65 (Cape Dorset, *Elkin and Bethke, 1995*)	NM within range

		116.21 (General, *Gamberg (unpublished data)*)			29.57 (Lake Harbour, *Elkin and Bethke, 1995*)<br>22.80 (Porcupine, *Gamberg, 2013*)<br>22.00 (Qamanirjuac, *Gamberg, 2013*)<br>24.95 (General, *Gamberg (unpublished data)*)<br>24.3 (Porcupine, *Gamberg et al., 2020*)	
Fe	470.30(295.61)	1594.97 (Bathurst, *Elkin and Bethke, 1995*)<br>701.84 (Arviat, *Elkin and Bethke, 1995*)<br>3627.66 (Cape Dorset, *Elkin and Bethke, 1995*)<br>3956.18 (Lake Harbour, *Elkin and Bethke, 1995*)<br>1065.15 (General, *Gamberg (unpublished data)*)	NM Lower*	192.12(89.55)	237.26 (Bathurst, *Elkin and Bethke, 1995*)<br>217.67 (Arviat, *Elkin and Bethke, 1995*)<br>194.94 (Southampton, *Elkin and Bethke, 1995*)<br>440.85 (Cape Dorset, *Elkin and Bethke, 1995*)<br>342.99 (Lake Harbour, *Elkin and Bethke, 1995*)<br>217.69 (General, *Gamberg (unpublished data)*)	NM Lower
Pb	0.06(0.08)	0.38 (Bathurst, *Elkin and Bethke, 1995*)<br>0.25 (Arviat, *Elkin and Bethke, 1995*)<br>2.64 (Cape Dorset, *Elkin and Bethke, 1995*)<br>3.38 (Lake Harbour, *Elkin and Bethke, 1995*)<br>0.89 (Leaf River, *Robillard et al.,2002*)<br>0.89 (George River, *Robillard et al., 2002*)<br>0.73 (General, *Gamberg (unpublished data)*)	NM Lower*	1.95(12.45)	0.11 (Bathurst, *Elkin and Bethke, 1995*)<br>0.10 (Arviat, *Elkin and Bethke, 1995*)<br>0.33 (Southampton, *Elkin and Bethke, 1995*)<br>0.42 (Cape Dorset, *Elkin and Bethke, 1995*)<br>0.47 (Lake Harbour, *Elkin and Bethke, 1995*)<br>0.28 (Leaf River, *Robillard et al.,2002*)<br>0.20 (George River, *Robillard et al., 2002*)<br>0.98 (Banks Island, *Larter and Nagy, 2000*)<br>0.21 (Bluenose, *Larter and Nagy, 2000*)<br>0.09 (Porcupine, *Gamberg, 2013*)	NM Higher

					0.30 (Qamanirjuac, *Gamberg, 2013*)<br>1.20 (General, *Gamberg (unpublished data)*)	
Mn	6.47(3.59)	12.62 (Bathurst, *Elkin and Bethke, 1995*)<br>10.84 (Arviat, *Elkin and Bethke, 1995*)<br>8.60 (Cape Dorset, *Elkin and Bethke, 1995*)<br>15.85 (Lake Harbour, *Elkin and Bethke, 1995*)<br>10.54 (General, *Gamberg (unpublished data)*)	NM Lower*	5.84(2.44)	8.96 (Bathurst, *Elkin and Bethke, 1995*)<br>12.03 (Arviat, *Elkin and Bethke, 1995*)<br>9.73 (Southampton, *Elkin and Bethke, 1995*)<br>11.66 (Cape Dorset, *Elkin and Bethke, 1995*)<br>18.62 (Lake Harbour, *Elkin and Bethke, 1995*)<br>8.00 (General, *Gamberg (unpublished data)*)	NM Lower*
Mo	1.84(0.75)	2.64 (General, *Gamberg (unpublished data)*)	NM Lower*	0.91(0.33)	0.76 (General, *Gamberg (unpublished data*)	NM Higher*
Se	1.70(1.32)	0.52 (General, *Gamberg (unpublished data)*)	NM Higher*	4.92(0.69)	4.80 (Porcupine, *Gamberg, 2013*)<br>4.20 (Qamanirjuac, *Gamberg, 2013*)<br>3.73 (General, *Gamberg (unpublished data)*)<br>4.5 (Porcupine, *Gamberg et al., 2020*)	NM Higher
Zn	87.21(47.95)	114.11 (Bathurst, *Elkin and Bethke, 1995*)<br>92.27 (Arviat, *Elkin and Bethke, 1995*)<br>75.84 (Cape Dorset, *Elkin and Bethke, 1995*)<br>76.27 (Lake Harbour, *Elkin and Bethke, 1995*)<br>101.16 (General, *Gamberg (unpublished data)*)	NM within range	127.91(16.14)	123.49 (Bathurst, *Elkin and Bethke, 1995*)<br>120.86 (Arviat, *Elkin and Bethke, 1995*)<br>111.87 (Southampton, *Elkin and Bethke, 1995*)<br>106.73 (Cape Dorset, *Elkin and Bethke, 1995*)<br>96.75 (Lake Harbour, *Elkin and Bethke, 1995*)<br>107.80 (Porcupine, *Gamberg, 2013*)<br>107.90 (Qamanirjuac, *Gamberg, 2013*)<br>114.00 (General, *Gamberg (unpublished data*)	NM Higher

* = Northern Mountain caribou mean element concentration (this study) is significantly different than ***all*** available means from other Canadian herds, at $p < 0.05/m$, where m is the number of 'other herd' means being tested against for each element (i.e. Bonferroni correction applied due to multiple comparisons).

References

1. Elkin BT and Bethke RW. Environmental contaminants in caribou in the Northwest Territories, Canada. Sci Tot Environ. 1995; 106/161:307-321. [Supplementary Table]
2. Gamberg M. Arctic Caribou Contaminant Monitoring Program. In: Smith S, Stow J, Edwards J, editors. Synopsis of Research Conducted under the 2013-2014 Northern Contaminants Program. 2013. pp. 291-298. [Supplementary Table]
3. Robillard S, Beauchamp G, Paillard G, and Bélanger D. Levels of cadmium, lead, mercury, and ^{137}caesium in caribou (*Rangifer tarandus*) tissues from northern Québec. Arctic. 2002; 55(1):1-9. [Supplementary Table]
