# Supplementary figures and images for: Evaluating the use of hair as a non-invasive indicator of trace mineral status in woodland caribou (Rangifer tarandus caribou)
Source: PLoS One. 2022 Jun 28;17(6):e0269441. doi: 10.1371/journal.pone.0269441 (PMC9239472; doi:10.1371/journal.pone.0269441)

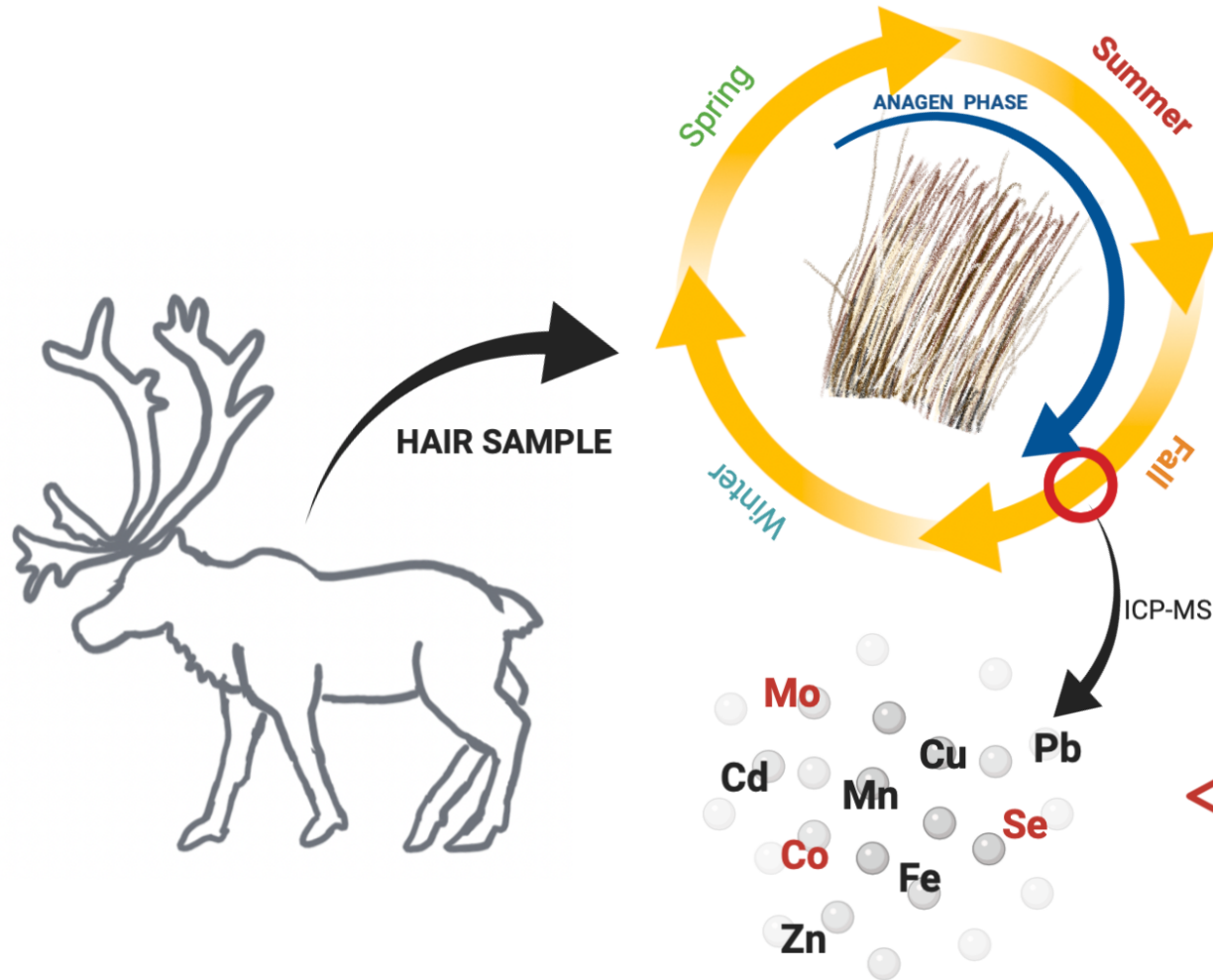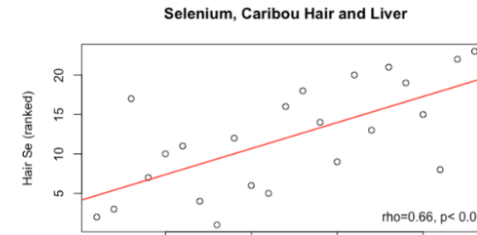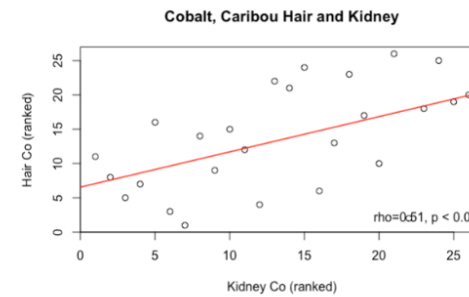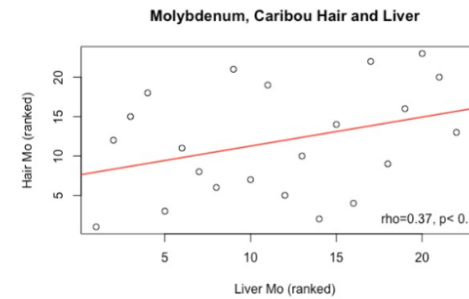

**Caribou Hair**  
=  
effective bioindicator for  
**Se status** ± other trace  
minerals/ heavy metals

Supplement: S1 Graphical abstract — (PDF) [file pone.0269441.s002.pdf]
